# Post-Kala-Azar Dermal Leishmaniasis in Mymensingh, Bangladesh

**DOI:** 10.4269/ajtmh.2011.11-0128

**Published:** 2011-08-01

**Authors:** Shamim Islam, M. Ashraful Alam Bhuiyan, Caryn Bern

**Affiliations:** Children's Hospital and Research Center Oakland, Oakland, California; ICDDR,B, Dhaka, Bangladesh; Division of Parasitic Diseases and Malaria, Center for Global Health, Centers for Disease Control and Prevention, Atlanta, Georgia

**Figure 1.**
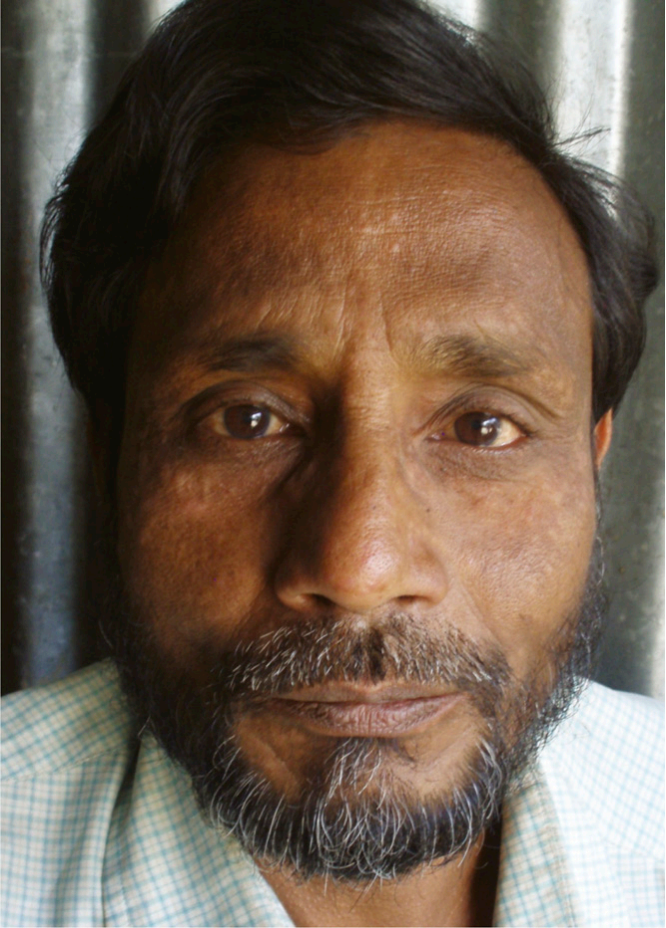
The patient has hypopigmented papules and plaques scattered over the face, most concentrated peri-orally and on the cheeks, common areas of involvement for post-kala-azar dermal leishmaniasis (PKDL). The patient consented to having his picture published in the journal.

**Figure 2.**
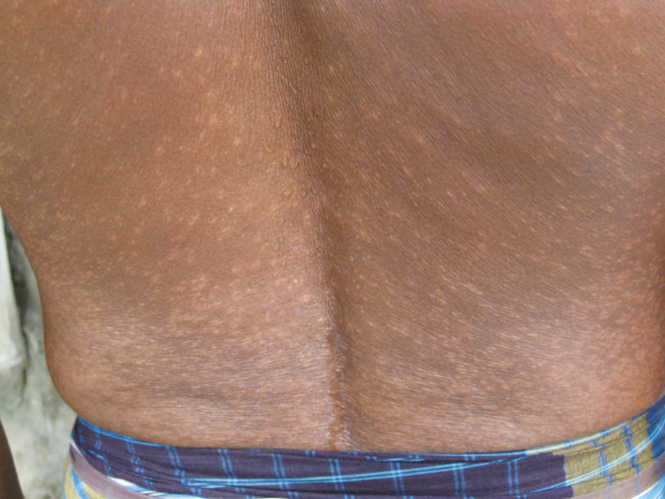
Hypopigmented papules are seen on the patient's back. The lesions are roughly symmetrical, a common characteristic of post-kala-azar dermal leishmaniasis (PKDL), and are painless and non- pruritic.

**Figure 3.**
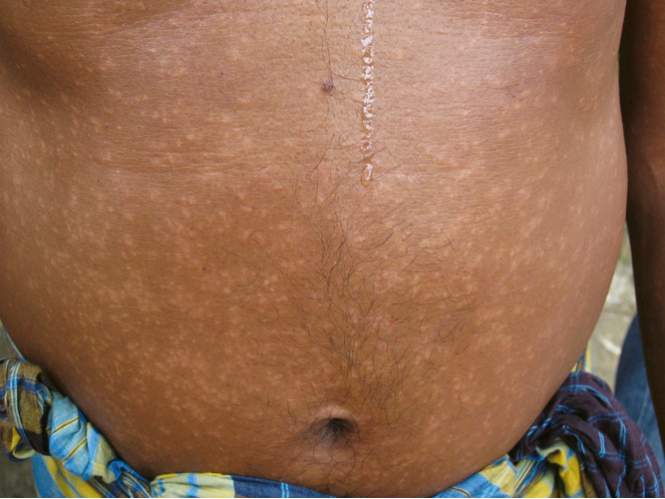
Hypopigmented papules on the abdomen.

**Figure 4.**
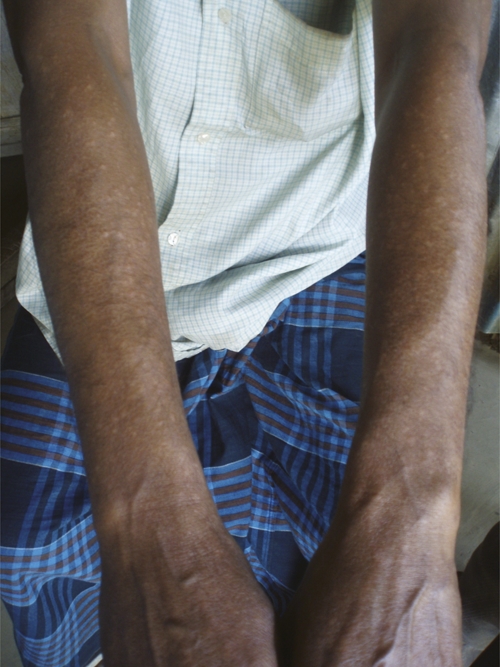
The patient has hypopigmented papules and plaques on both forearms. The absence of sensorineural changes helps to distinguish post-kala-azar dermal leishmaniasis (PKDL) from leprosy, which commonly presents with hypopigmented macules or patches associated with hypoesthesia or anesthesia, and neural thickening.

A 50-year-old man from Mymensingh district, Bangladesh, presented with a 3-month history of non-pruritic, painless hypopigmented papules, and plaques, beginning on the face and subsequently spreading to the forearms, torso, and legs (Figures 1–4) Fifteen months before the onset of skin lesions, the patient had visceral leishmaniasis (kala-azar), successfully treated with 30 intramuscular injections of sodium stibogluconate (SSG). Polymerase chain reaction showed *Leishmania donovani* DNA in a buffy coat specimen.

Post-kala-azar dermal leishmaniasis (PKDL) is a chronic skin rash usually seen in apparently cured kala-azar patients in East Africa and South Asia. In the Indian subcontinent, the cumulative PKDL incidence after kala-azar is 5–10%.^1^ The PKDL usually presents with erythematous or hypopigmented papules or macules, sometimes progressing to plaques or nodules. Kala-azar patients treated with antimonial drugs may have poorer immune recovery and a higher risk of subsequent PKDL compared with those treated with amphotericin formulations.^2^ The only PKDL treatment regimens with proven efficacy in South Asia consist of 120 SSG injections over 6 months or miltefosine 2.5 mg/kg/day for 12 weeks. The PKDL patients are infectious to the sand fly vector and are thought to represent an important parasite reservoir in the anthroponotic transmission cycle of visceral leishmaniasis in South Asia.^1^
